# Variation in fine‐scale genetic structure and local dispersal patterns between peripheral populations of a South American passerine bird

**DOI:** 10.1002/ece3.3342

**Published:** 2017-09-08

**Authors:** Esteban Botero‐Delgadillo, Verónica Quirici, Yanina Poblete, Élfego Cuevas, Sylvia Kuhn, Alexander Girg, Kim Teltscher, Elie Poulin, Bart Kempenaers, Rodrigo A. Vásquez

**Affiliations:** ^1^ Instituto de Ecología y Biodiversidad Departamento de Ciencias Ecológicas Facultad de Ciencias Universidad de Chile Santiago Chile; ^2^ SELVA: Research for conservation in the Neotropics Bogotá Colombia; ^3^ Departamento de Ecología y Biodiversidad Facultad de Ecología y Recursos Naturales Universidad Andrés Bello Santiago Chile; ^4^ Centro de Investigación Para la Sustentabilidad Universidad Andrés Bello Santiago Chile; ^5^ Instituto de Ciencias Naturales Universidad de las Américas Santiago Chile; ^6^ Doctorado en Medicina de la Conservación Facultad de Ecología y Recursos Naturales Universidad Andrés Bello Santiago Chile; ^7^ Department of Behavioural Ecology and Evolutionary Genetics Max Plank Institute for Ornithology Seewiesen Germany

**Keywords:** breeding dispersal, capture‐mark‐recapture, fine‐scale genetic structure, intraspecific variation, natal dispersal, thorn‐tailed rayadito

## Abstract

The distribution of suitable habitat influences natal and breeding dispersal at small spatial scales, resulting in strong microgeographic genetic structure. Although environmental variation can promote interpopulation differences in dispersal behavior and local spatial patterns, the effects of distinct ecological conditions on within‐species variation in dispersal strategies and in fine‐scale genetic structure remain poorly understood. We studied local dispersal and fine‐scale genetic structure in the thorn‐tailed rayadito (*Aphrastura spinicauda*), a South American bird that breeds along a wide latitudinal gradient. We combine capture‐mark‐recapture data from eight breeding seasons and molecular genetics to compare two peripheral populations with contrasting environments in Chile: Navarino Island, a continuous and low density habitat, and Fray Jorge National Park, a fragmented, densely populated and more stressful environment. Natal dispersal showed no sex bias in Navarino but was female‐biased in the more dense population in Fray Jorge. In the latter, male movements were restricted, and some birds seemed to skip breeding in their first year, suggesting habitat saturation. Breeding dispersal was limited in both populations, with males being more philopatric than females. Spatial genetic autocorrelation analyzes using 13 polymorphic microsatellite loci confirmed the observed dispersal patterns: a fine‐scale genetic structure was only detectable for males in Fray Jorge for distances up to 450 m. Furthermore, two‐dimensional autocorrelation analyzes and estimates of genetic relatedness indicated that related males tended to be spatially clustered in this population. Our study shows evidence for context‐dependent variation in natal dispersal and corresponding local genetic structure in peripheral populations of this bird. It seems likely that the costs of dispersal are higher in the fragmented and higher density environment in Fray Jorge, particularly for males. The observed differences in microgeographic genetic structure for rayaditos might reflect the genetic consequences of population‐specific responses to contrasting environmental pressures near the range limits of its distribution.

## 1 INTRODUCTION

Fine‐scale genetic population structure arises when alleles or genotypes are nonrandomly distributed across space. This is typically a consequence of the interaction between environmental heterogeneity and species‐specific life history traits such as dispersal patterns, mating systems, and demography (Garroway et al., 2013; Greenwood & Harvey, 1982; Lee, Simeoni, Burke, & Hatchwell, 2010; Van Dijk, Covas, Doutrelant, Spottiswoode, & Hatchell, 2015; Woxvold, Adcock, & Mulder, 2006). Even though mobile organisms such as birds are expected to show high levels of gene flow within and among populations, spatial genetic structure has been detected in a wide variety of species with differing dispersal abilities and social organization (e.g., Beck, Peakall, & Heinsohn, 2008; Browne, Collins, & Anderson, 2008; Klauke, Schaefer, Bauer, & Segelbacher, 2016; Lee et al., 2010; Pierson, Allendorf, Saab, Drapeau, & Schwartz, 2010; Temple, Hoffman, & Amos, 2006).

Studies examining fine‐scale genetic structure in birds have been carried out at different spatial scales, depending on the underlying factors and hypotheses being considered. Several studies focusing on the influence of habitat heterogeneity on gene flow have investigated spatial variation at the landscape level (>10 km), and detected genetic differences among populations of species that showed high habitat specificity (e.g., Browne et al., 2008; Walsh, Kovach, Babbit, & O′Brien, 2012; Woltmann, Kreiser, & Sherry, 2012) or sex‐biased and/or restricted dispersal (e.g., Coulon, Fitzpatrick, Bowman, & Lovette, 2010; Coulon et al., 2008; Hermes, Döpper, Schaefer, & Segelbacher, 2016; Pierson et al., 2010). On the other hand, studies aiming at linking spatial patterns to complex social interactions have assessed genetic variation at a smaller geographic scale (<5 km), and found fine‐scale genetic structure within populations of cooperatively breeding and lekking birds (e.g., Beck et al., 2008; Double, Peakall, Beck, & Cockburn, 2005; Van Dijk et al., 2015).

Although complex social interactions are viewed as a main driver of fine‐scale genetic structure within local populations of birds (Hatchwell, 2009; Van Dijk et al., 2015), environmental heterogeneity can also affect the distribution of individuals at small spatial scales (Lindsay et al., 2008), especially if nonmigratory movements are restricted by habitat patchiness (Harris & Reed, 2002). Natal and breeding dispersal can be severely limited even if there are only relatively small gaps between patches of suitable habitat. For example, some nonmigratory forest‐specialists, particularly small insectivorous passerines, seem reluctant to cross 50–200 m open areas (Baguette, Legrand, Fréville, van Dyck, & Ducatez, 2012; Moore, Robinson, Lovette, & Robinson, 2008; Sieving, Willson, & De Santo, 1996; Van Houtan, Pimm, Halley, Bieregaard, & Lovejoy, 2007). This means that a heterogeneous distribution of suitable habitat can produce strong microgeographic genetic structure, but this has seldom been assessed (Walsh et al., 2012). Furthermore, given that the effects of environmental heterogeneity and demographic processes on bird movements can differ between females and males, patterns of local dispersal and fine‐scale genetic structure can be sex‐specific (Double et al., 2005; Pierson et al., 2010).

Dispersal behavior can also vary spatially due to local differences in habitat heterogeneity, demographic factors, and social interactions (Bowler & Benton, 2005; Cote & Clobert, 2012), and hence, intraspecific variation in dispersal and in population spatial structure is expected under distinct environmental contexts (see Matthysen, 2012). As variability in habitat patchiness and population density can affect local gene flow (Walsh et al., 2012), distinct patterns of fine‐scale genetic structure between breeding populations are likely to occur (Hermes et al., 2016; Lee et al., 2010; Stow, Sunnucks, Briscoe, & Gardner, 2001). However, the mechanisms behind within‐species variation in dispersal patterns and in microgeographic genetic structure and the effects of varying life histories or contrasting ecological conditions remain relatively poorly understood (Le Galliard, Massot, & Clobert, 2012; Van Dijk et al., 2015).

**Figure 1:**
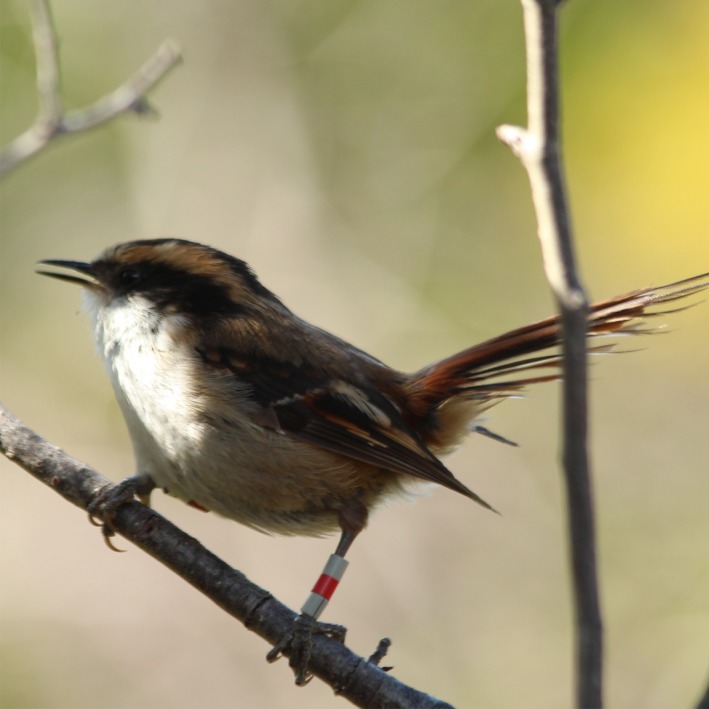
Color‐banded adult thorn‐tailed rayadito *Aphrastura spinicauda* (Photo: Silvia Lazzarino, reproduced with permission)

Here, we combine capture‐mark‐recapture (CMR) data and molecular genetics to examine local natal and breeding dispersal and fine‐scale genetic structure in two peripheral populations of a South American forest bird, the thorn‐tailed rayadito *Aphrastura spinicauda* (Furnariidae; Figure 1) that inhabit contrasting environments in southern and north‐central Chile. Comparisons between disconnected populations facing distinct environmental pressures (e.g., Spinks, Jarvis, & Bennett, 2000) are interesting models to assess intraspecific variation in dispersal behavior and its potential consequences (Foster, 1999; Hampe & Petit, 2005; Merrick & Koprowski, 2016). Moreover, comparing localities at distributional range limits (i.e., peripheral localities) can provide insight into population‐specific responses to environmental changes (Le Galliard et al., 2012; Travis & Dytham, 2012).

**Figure 2:**
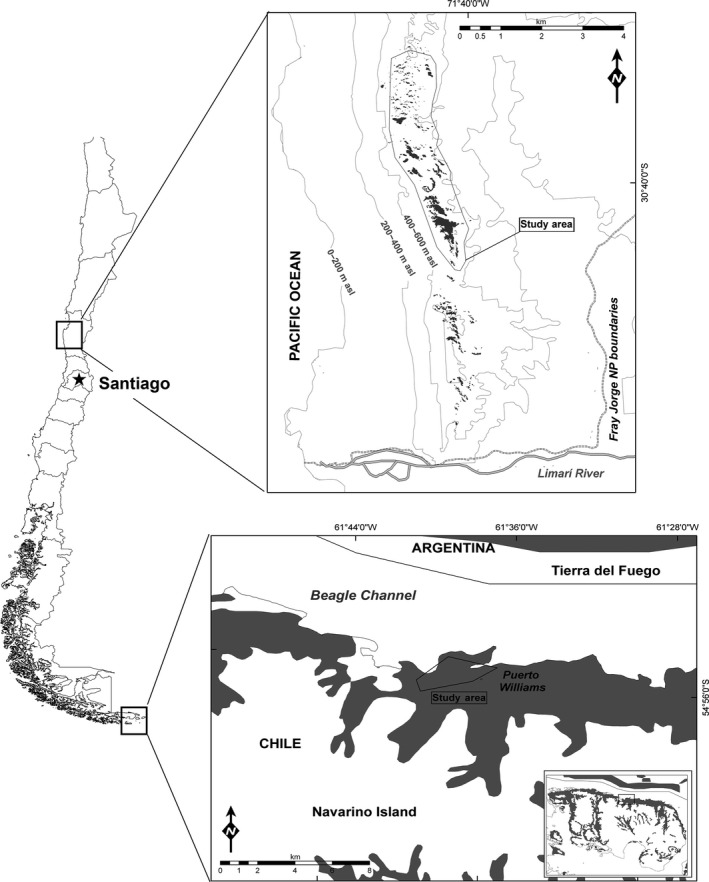
Location of the two study sites in two peripheral populations of thorn‐tailed rayadito, Fray Jorge National Park, north‐central Chile (top), and Navarino Island, southern Chile (bottom). Gray areas in both panels represent suitable forest breeding habitat

The thorn‐tailed rayadito is a secondary cavity nesting bird distributed along an extensive latitudinal gradient (30°S–55°S; Figure 2) in Chile and Argentina (Remsen, 2006). Despite the astonishing behavioral and ecological diversity of the Furnariidae, there is a paucity of information on the spatial ecology and population dynamics for almost all members of this family (see Remsen, 2006). Rayaditos are socially monogamous, and both sexes incubate the eggs and share nestling provisioning equally (Moreno, Merino, Lobato, Rodríguez‐Gironés, & Vásquez, 2007; Moreno, Merino, Vásquez, & Armesto, 2005). There is no information regarding sex differences in territory establishment and nest‐site selection, but it is expected that both sexes are involved as seems to be the case in other furnariids (Remsen, 2006). Breeding appears to be limited by the availability of natural cavities in secondary‐growth and human‐altered landscapes (Cornelius, 2008; Tomasevic & Estades, 2006), and populations are negatively affected by forest fragmentation at a local scale (Vergara & Marquet, 2007). Although the species has colonized islands located up to 80–100 km from the continent, rayaditos are rather sedentary and seldom fly distances over 300 m to cross open areas or unsuitable habitat (Vergara, Hahn, Zevallos, & Armesto, 2010).

In this study, we focused on two populations of rayadito located on opposite ends of the species' breeding range (Figure 2) and differing in habitat heterogeneity, population density, and levels of stress (Quirici, Guerrero, Krause, Wingfield, & Vásquez, 2016; Quirici et al., 2014). The first population is located on Navarino island (55°4′S, 67°40′W), southern Chile (hereafter Navarino or Nav), which represents a continuous and less populated environment; the second population is located in Fray Jorge National Park (30°38′S, 71°40′W), north‐central Chile (hereafter Fray Jorge or FJ), which represents a fragmented and densely occupied habitat. We used eight years of CMR data to investigate (1) whether natal and breeding dispersal vary between these contrasting environments and (2) whether there is a correspondence between local dispersal patterns and population‐specific fine‐scale genetic structure.

## 2 MATERIALS AND METHODS

### 2.1 Study populations

This study is part of a long‐term research project on the breeding ecology of the thorn‐tailed rayadito in Navarino and Fray Jorge, where 171–222 and 101–157 nest boxes have been available in 2006–2015, respectively. Nest boxes were systematically distributed in second‐growth forests in Navarino (24 ± 0.6 m between nearest nest boxes; mean ± *SE*) and in forest relicts in Fray Jorge (21 ± 2.6 m between nearest nest boxes). The position of all nest boxes was georeferenced with a 2 m measurement error. The spatial arrangement of the nest boxes was defined after considering previous density estimates of breeding birds (see e.g., Vergara & Marquet, 2007).

The study area at Navarino covers ca. 4.3 km^2^ in a landscape dominated by sub‐Antarctic forests (Rozzi & Jiménez, 2014), with woodland habitat comprising ca. 3.3 km^2^ (Figure 2). The study area at Fray Jorge is located in a coastal mountain range reaching up to 630 m a.s.l. and covers ca. 5.3 km^2^ in a predominantly semiarid landscape, dominated by matorral steppe (Luebert & Pliscoff, 2006), with a relict forest composed of several fragments (del‐Val et al., 2006; Figure 2), ranging in size between 0.5 and 22.5 ha and covering a total area of 2.4 km^2^ (Cornelius, Cofré, & Marquet, 2000).

While birds from Navarino have maintained recurrent gene flow with other rayadito populations, the Fray Jorge population is isolated (González & Wink, 2010; Yáñez, 2013). Bird density estimates during the breeding season based on distance sampling indicate that Fray Jorge is almost three times more densely populated (8.2 pairs/ha; Vergara & Marquet, 2007) than Navarino (2.9 pairs/ha; Botero‐Delgadillo, 2017). Similarly, nest box occupation rates during 2008–2015 were higher for Fray Jorge (18%) than for Navarino (10%; data from this study). Measurements of baseline corticosterone levels in blood plasma from breeding adults were also significantly higher in Fray Jorge (2010: 0.79 ± 0.11 ng/ml; 2011: 0.92 ± 0.05 ng/ml) than in Navarino (2010: 0.49 ± 0.12 ng/ml; 2011: 0.83 ± 0.09 ng/ml), suggesting that Fray Jorge is a more stressful environment (Quirici et al., 2014, 2016).

### 2.2 Field methods

Data for our study were obtained during eight consecutive breeding seasons (2008–2015) in both localities. Adults were captured inside the nest boxes when feeding 12‐ to 14‐day‐old nestlings using a manually triggered trap (see details in Moreno et al., 2005; Quirici et al., 2014). Adults and nestlings were marked with a uniquely numbered metal ring, and blood sampled (ca. 15 μl) by puncturing the brachial vein with a sterile needle (Quirici et al., 2014). Blood samples were stored on filter paper (FTA™ Classic Cards, Whatman™) for subsequent genetic analyzes. Dispersal events were identified based on recaptures of individuals in subsequent years.

### 2.3 Data analysis—dispersal estimates and CMR data

All analyzes of natal and breeding dispersal were based on CMR data obtained during 2008–2015. Default options were used to define parameter values in all analyzes, unless otherwise stated in the text or in the corresponding references. Natal dispersal was defined as the movement between the natal nest box and the first recorded breeding box, while breeding dispersal comprised movements between consecutive breeding seasons. In order to characterize dispersal behavior, we calculated the frequency of dispersal (% of dispersing birds) and the distance moved (m) by recaptured individuals (Clarke, Sæther, & Røskaft, 1997), and also analyzed the spatial patterns of bird movements.

For calculating dispersal frequency, we first applied Dirichlet tessellation (Aurenhammer, 1991) to model the territories of all breeding pairs in both localities for every year (2008–2015), using the spatial patterns of nest box occupation as input data (Adams, 2001; Valcu & Kempenaers, 2008). Mean territory diameters were subsequently calculated for each year/population, and individuals were considered as “dispersed” if they moved over a distance equal or longer than the upper limit of the 95% confidence interval (CI) of the annual mean territory diameter (Valcu & Kempenaers, 2008); individuals that moved shorter distances or that remained in their natal or previous breeding site were categorized as “nondispersed.” Dirichlet tessellation and estimation of territory diameters were performed in the Analysis tools in ArcGIS 9.3 (ESRI, Redlands, CA, USA).

For describing spatial patterns of dispersal, we used descriptive analyzes and performed statistical tests with R version 3.3.1 (R Development Core Team), using α = .05 for hypothesis testing. To compare natal and breeding dispersal distances between the sexes in each population we employed Kolmogorov–Smirnov tests (see Harvey, Greenwood, Campbell, & Stenning, 1984; Harvey, Greenwood, & Perrins, 1979). Because some breeding adults were recaptured more than once, K‐S tests for breeding dispersal were performed on two datasets: a first reduced set comprising only the first breeding dispersal distance recorded for every individual, and a second, expanded set, including all distances from some repeatedly recaptured birds (Montalvo & Potti, 1992). Subsequently, we used randomization tests to evaluate the spatial patterns of natal and breeding dispersal for each sex and population, comparing the observed median dispersal distances against null distributions of medians generated by 1,000 Monte Carlo simulations to calculate exact probabilities. Simulated distances were obtained by assigning each breeding individual to any of the “available” nest boxes during the year of its recapture, with none of them being assigned more than once in every run (Wheelwright & Mauck, 1998). We tested two null models that varied in the probability of assignment of the “available” nest boxes for each individual: The first was used for testing if median distances departed from a completely random pattern, using a null uniform distribution for the probability of assignment (i.e., probability of assignment was equal for all nest boxes); the second was used to test if median distances departed from a random‐walk search, using an exponential distribution for the probability of assignment that varied as a function of the distance from the natal site (Waser, 1985; Winkler et al., 2005). Exponential null distributions were simulated for each locality based on the regression of the probability of recapture on distance (Winkler et al., 2005). None of these models assumed age‐related social dominance (Wheelwright & Mauck, 1998). All randomization tests were run with the coin package in R (Hothorn, Hornik, van de Wiel, & Zeiles, 2008).

We also tested for population differences in sex‐specific dispersal patterns, using generalized linear mixed models (GLMM) to compare natal/breeding dispersal distances (m). Given that density of breeding pairs, and therefore, territory size, can vary between populations and years, we did not use the distances moved by dispersing birds in these analyzes as they might not be directly comparable. Instead, we transformed distances into territory units (Arcese, 1989), using the mean territory diameter estimated for every year and for each study site. For the natal dispersal analysis, the full model included sex, population of origin and their interaction as fixed effects, and the natal year and the time interval for recapture (not all nestlings were recaptured the following year) as random effects. The full model for breeding dispersal included the same fixed effects, and as random effects we added year of recapture, minimum estimated age (ages for adults were not always certain), and bird identity, given that many individuals were recaptured in multiple years. We fitted negative binomial models with a log‐link function using the lme4 (Bates, Maechler, Bolker, & Walker, 2015) package in R. We assessed goodness of fit of a set of candidate models and selected a minimal most adequate model based on values of the corrected Akaike's Information Criterion AICc (Crawley, 1993) using the MuMIn package (Bartoń, 2000).

Log‐linear analyzes were used to test whether the frequency of dispersers and nondispersers was independent from bird sex and population of origin. Because virtually all individuals showed natal dispersal (see Results), this analysis was carried out only for breeding dispersal. We used a backward hierarchical method and log‐likelihood ratio tests to obtain a minimal most adequate model from a full model that included dispersal status, population of origin, sex, and their interactions as factors.

Finally, to test the critical assumption that dispersal patterns were not affected by sex differences in mortality rates or probability of recapture (Ward & Weatherhead, 2005), we used two different approaches. For adult birds, we used CMR data to calculate apparent survival probability (φ) and recapture rate (p) using the Cormack–Jolly–Seber model (Sandercock, 2003) as implemented in MARK 5.1 (Cooch & White, 2008; White & Burnham, 1999). We generated different candidate models with different restrictions on the parameters (Lebreton, Burnham, Clobert, & Anderson, 1992), and used AICc values for model selection (see Table S2). For fledgling survival, we had to use sex‐specific recovery rates as an approximation of survival given the low recapture rates (see Results). We corroborated the validity of this approach by calculating the percentage of fledglings recaptured during our study in relation to an estimation of all surviving postfledging birds in each locality. This was done by first building a static life table (Gotelli, 2008), using CMR data to compute age‐specific survival and fecundity (see Table S3). Then, assuming no population increase/decrease during the study period, we calculated fledgling survival rates needed to maintain both populations constant (i.e., *r *=* *0; see Table S3). The ratio of recaptured/surviving nestlings was interpreted as a rough estimate of how representative the recapture rates were.

### 2.4 Genetic analyzes

DNA was extracted from blood samples using a QIAamp^®^ DNA Micro Kit (QIAGEN^®^ #56304). Given the absence of evident sexual dimorphism in rayaditos (Moreno et al., 2007), sex was determined using one chromosome‐linked marker (P2/P8; Griffiths, Double, Orr, & Dawson, 1998). All individuals were genotyped at 13 polymorphic microsatellite loci (see Table S1): species‐specific markers As1, As7, As18, As25‐1, As25‐5, As25‐8, As25‐10, and As25‐14 (Yáñez, Quirici, Castaño‐Villa, Poulin, & Vásquez, 2015), and the cross‐species amplifying markers Asμ15_ZEST, CcaTgu23 (Olano‐Marín et al., 2010), Tgu06 (= CK307697), Tgu05 (= DV946651) (Slate, Hale, & Birkhead, 2007), and ZF_AC138573 (Van Oosten, Mueller, Ottenburghs, Both, & Kempenaers, 2016).

Microsatellite amplifications were performed in multiplex PCRs using the Type‐it^®^ Microsatellite PCR Kit (QIAGEN^®^ #206246) and primer mixes containing four to five primer pairs (mix 1, 2, and 3; see Table S1). The forward primer of each pair was fluorescently labeled with 6‐FAM^™^, VIC^®^, PET^®^, or NED™ (Dye Set G5; Thermo Fisher Scientific). Primer concentrations in these mixes were adapted due to differences in amplification efficiency and dye strength (see Table S1). Each 10 μl multiplex PCR contained 15–80 ng DNA, 5 μl of the 2x Type‐it^®^ Microsatellite PCR Master Mix, and 1 μl of one of a primer mix. Cycling conditions were as follows: 5 min initial denaturation at 95°C, 23 (mix1 and 2) or 28 cycles (mix 3) of 30 s denaturation at 94°C, 90 s annealing at the temperature given in Table S1, and 1 min extension at 72°C, followed by a 30 min completing final extension at 60°C. After amplification, 1.5 μl of the PCR products was added to 13 μl formamide containing the GeneScan™ 500 LIZ^®^ Size Standard, heat denatured and resolved in POP4 polymer on an ABI™ 3130 Genetic Analyzer (Thermo Fisher Scientific, Darmstadt, Germany). Raw data were analyzed and alleles assigned using the genemapper 4.0 software (Applied Biosystems).

### 2.5 Data analysis – spatial genetic structure

All genetic analyzes were based on genotyped breeding adults that were captured during 2010–2015. We tested for linkage disequilibrium separately on datasets for each population, using exact tests in GENEPOP 4.0 (Raymond & Rousset, 1995). Tests for deviations from Hardy–Weinberg equilibrium (HWE) and estimation of frequency of null alleles were applied on the same datasets in CERVUS 3.0.7 (Kalinowski, Taper, & Marshall, 2007). There was no evidence of linkage disequilibrium between any pair of loci. With the exception of marker As1 (not included in further analyzes), no significant deviations from HWE were detected after applying Bonferroni correction for multiple comparisons (all *p *>* *.1), and frequency of null alleles never exceeded 0.05. Details on genetic diversity measures are addressed elsewhere (see Yáñez et al., 2015).

We evaluated population patterns of fine‐scale genetic structure in three ways: (1) spatial autocorrelation analyzes and heterogeneity tests were applied to investigate if local spatial genetic structure in each population corresponded to dispersal patterns observed with CMR data; (2) two‐dimensional autocorrelation analyzes (2D LSA) were implemented to explore how spatial genetic autocorrelation was distributed in a two‐dimensional landscape; and (3) Mantel tests (Mantel, 1967) of matrix correspondence were used to evaluate if patterns of isolation by distance were present at the local scale.

Separate spatial autocorrelation analyzes were performed for each sex/population (Nav: 82 females, 79 males; FJ: 69 females, 66 males) based on all genotyped individuals for 2010–2015 (e.g., Van Dijk et al., 2015). We repeated the same analyzes on “snapshot” years to check for possible temporal sampling effects due to demographic processes (Foerster, Valcu, Johnsen, & Kempenaers, 2006; Van Dijk et al., 2015), selecting the year with the largest number of captured/recaptured adults (Nav: 2014, *n *=* *63; FJ: 2013, *n *=* *50). Spatial autocorrelation analyzes were conducted in genalex 6.5 (Peakall & Smouse, 2012), following the methods described by Peakall, Ruibal, and Lindenmayer (2003) and Banks and Peakall (2012). For calculating genetic correlation coefficients (*r*), genalex uses pairwise squared genetic and pairwise geographic distance matrices, and then plots *r‐*values as a function of distance across different distance class intervals (Peakall, Smouse, & Hubb, 1995). For the geographic distance matrix, UTM coordinates from nest boxes were entered as input data. Given the difficulties to define a representative distance that could reveal the extent of nonrandom genetic structure, we calculated *r* for increasing distance classes using the Multiple Dclass option in genalex to define the first distance interval for traditional correlograms (Peakall et al., 2003). We explored several distance classes ranging from the overall mean territory diameters in Navarino and Fray Jorge (100 and 50 m, respectively) to the maximum distance between nest boxes in each locality (2.9 and 4.2 km). The minimum distance class size for which *r* was significant (150 m; see Results) was subsequently used as the distance interval for traditional correlograms (Peakall et al., 2003). Significance of positive/negative autocorrelation values for traditional and Multi Dclass analyzes was assessed by estimating the 95% CI about *r* using bootstrap resampling, and also through 1000 random permutations of bird genotypes to generate estimates of *r* that would be obtained randomly (*r*_*p*_). We rejected the null hypothesis of no autocorrelation when both estimates indicated significant nonrandom genetic structure (i.e., the 95% CI around *r* did not include 0 and *r *>* *95% CI around *r*_*p*_). Although this is a conservative approach, it is highly recommended when sample sizes are relatively small (Peakall et al., 2003). Nonparametric heterogeneity tests were applied for testing sex differences in the correlograms by computing the *t* ^2^ statistic for each distance class and the correlogram wide “Omega” (ω) (see Banks & Peakall, 2012; Smouse, Peakall, & González, 2008).

The 2D LSAs were performed separately for each sex/population, estimating the local genetic correlation (*lr*) based on pairwise comparisons between every individual and its *n* nearest neighbors. Calculation of *lr* and standard permutation tests (1,000 permutations/run) for significance was conducted in genalex as described by Double et al. (2005). We used multiple runs calculating *lr* for four, nine, and 14 nearest neighbors (i.e., subsets of five, 10 and 15 individuals), but only presented results for 14 nearest neighbors, as such subsets comprised an equivalent linear distance to the distance class size used for spatial autocorrelation analyzes (see Results). Mantel tests were also implemented in genalex, using permutation tests (1,000 permutations) to assess the significance of correlations between matrices of genetic similarity and geographic distances (Peakall et al., 2003).

## 3 RESULTS

### 3.1 Natal dispersal patterns

**Figure 3:**
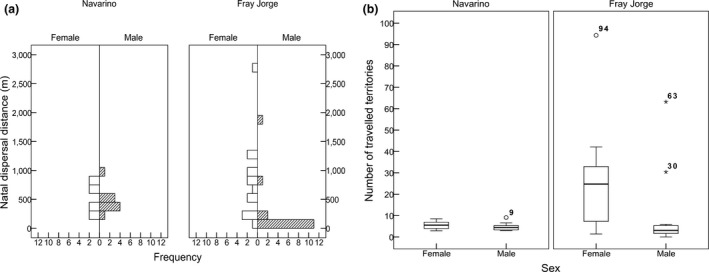
Natal dispersal patterns in two populations of thorn‐tailed rayadito based on capture‐mark‐recapture data obtained during 2008–2015. A total of 17 (females: 8, males: 9) and 29 (females: 14, males: 15) postfledging birds were recaptured in Navarino and Fray Jorge, respectively. (a) Frequency distribution of natal dispersal distances traveled by female and male birds in Navarino Island and Fray Jorge National Park. (b) Natal dispersal was compared between both populations after transforming distances into territory units based on Dirichlet tessellation for each breeding season

From a total of 565 fledglings marked in Navarino up to 2014, 17 (3%) were recaptured at least once as breeding adults, of which eight were females and nine were males. Twelve individuals were recaptured the year after hatching, the remainder after two (*n *=* *3) or 3 years (*n *=* *2). Based on the distance moved from their natal nest box, all 17 recaptured birds were defined as dispersers. The median distance moved by females (490 m, range: 180–760) and males (420 m, 190–840) was similar, and there was no sex‐difference in the distribution of traveled distances (Kolmogorov–Smirnov test: *p *=* *.46, *Z *=* *0.81, *n *=* *17; see Figure 3a). Monte Carlo simulations revealed that the median distance moved by either sex was not significantly different from median distances generated by the completely random (females: *p *=* *.44; males: *p *=* *.49) and random‐walk models (females: *p *=* *.21; males: *p *=* *.28).

From 491 marked fledglings in Fray Jorge up to 2014, 29 (6%) were recaptured as breeding adults, including 14 females and 15 males. Individuals were recaptured one (*n *=* *1), two (*n *=* *19), three (*n *=* *8), or four (*n *=* *1) years after hatching. Three males were recaptured in their natal box (nondispersed); all other cases were defined as dispersal events. Females traveled longer median distances (740 m, range: 40–2,730) than males (100 m, 0–1,830), and the distribution of movement distances differed significantly between the sexes (Kolmogorov‐Smirnov test: *p *<* *.001, *Z *=* *1.95, *n *=* *29; see Figure 3a). Monte Carlo simulations showed that median distances moved by females were not different from values generated by the completely random model, but distances moved by males were shorter than expected (females: *p *=* *.35; males: *p *=* *.041); on the other hand, the random‐walk model showed that females moved significantly longer distances than expected, but male movements were not different from values generated under the null hypothesis (females: *p *=* *.045; males: *p *=* *.58).

As expected based on differences in breeding density, adult birds in Navarino occupied larger territories (Dirichlet tiles) than those in Fray Jorge. The upper limit of the 95% CI of the mean territory diameter in Navarino ranged from 63 to 110 m across breeding seasons, while in Fray Jorge it ranged from 29 to 39 m. We also compared natal dispersal distances as territory units (tU). In Navarino, movements of females (median: 5 tU, range: 3–8) and males (4 tU, 3–9) were similar, while there was a marked difference between the sexes in Fray Jorge (females: 25 tU, 1–94; males: 3 tU, 0–60; Figure 3b). After correcting for random effects (natal year and interval of recapture), the most adequate model showed a significant sex × population effect on the number of territories moved by natal dispersers (Table 1).

**Table 1:** Best supported GLMMs for between‐population comparisons of natal and breeding dispersal distances of thorn‐tailed rayaditos

Dispersal type	Explanatory terms	Estimate	*SE*	Test statistic	*p*	Variance (%)
Natal dispersal	Intercept	3.31	0.39	*z*_45 _= 8.43	<.001	
	Sex*Population	1.34	0.65	*z*_42 _= 2.05	.04	
	Random effects					
	Natal year					7.66
	Recapture interval					28.65
Breeding dispersal	Intercept	−0.83	0.26	*z*_288 _= −3.24	.001	
	Sex	−0.35	0.35	*z*_287 _= −0.99	.32	
	Population	−0.18	0.37	*z*_286 _= −0.49	.62	
	Sex*Population	0.47	0.54	*z*_285 _= 0.88	.38	
	Random effects					
	Age					0
	Recapture year					0.05
	Bird identity					49.62

Parameter estimates and *SE* (standard errors) were estimated relative to “Female” for “Sex” and relative to “Fray Jorge” for “Population.” The *z* statistic corresponds to the Wald test. The proportion of variance explained by each random effect is also indicated.

### 3.2 Breeding dispersal patterns

Within‐population patterns of breeding dispersal were similar for the reduced and expanded datasets (see Methods), and therefore, we report descriptive and statistical analyzes incorporating all distances, including those from adults that were recaptured more than once (see Montalvo & Potti, 1992).

**Figure 4:**
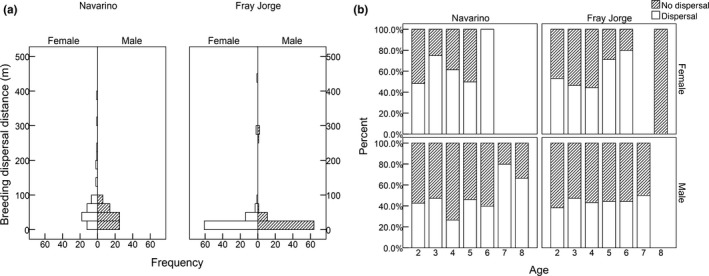
Breeding dispersal patterns in two populations of thorn‐tailed rayadito based on capture‐mark‐recapture data obtained during 2008–2015. A total of 127 (females: 57, males: 70) and 162 (females: 83, males: 79) recapture events were obtained in Navarino and Fray Jorge, respectively. (a) Frequency distribution of distances traveled by birds between consecutive breeding seasons in Navarino Island and Fray Jorge National Park. (b) Frequency of breeding dispersal events for female and male birds in Navarino and Fray Jorge according to their minimum estimated age

From a total of 210 captured adults in Navarino, we obtained 127 recapture events of 69 individuals between consecutive breeding seasons (57 recaptures of 35 females, 70 recaptures of 33 males). The distribution of traveled distances did not differ significantly between the sexes, even though females tended to move farther (females: median: 45 m, range: 0–390; males: 30 m, 0–90; Kolmogorov‐Smirnov test: *p *=* *.07, *Z *=* *1.23, *n *=* *127; see Figure 4a). Monte Carlo simulations suggested that median distances for females and males were shorter than expected under the completely random model (females: *p *=* *.039; males: *p *=* *.033), albeit not different from values taken from a random‐walk distribution (females: *p *=* *.64; males: *p *=* *.68).

From the 197 captured adults in Fray Jorge, 162 recaptures of 92 individuals were obtained between consecutive breeding seasons (83 recaptures of 50 females, 79 recaptures of 42 males). No difference in the distribution of traveled distances was observed between the sexes (females: median: 11 m, 0–270; males: 7 m, 0–270; Kolmogorov‐Smirnov test: *p *=* *.65, *Z *=* *0.56, *n *=* *162; see Figure 4a). As in the Navarino population, Monte Carlo simulations suggested that median dispersal distances were shorter than randomly generated distances in the two sexes (females, *p *=* *.028; males, *p *=* *.012), but not differing from values generated under the random‐walk model (females, *p *=* *.28; males, *p *=* *.37).

In terms of number of territories traveled, females (Navarino: 0.6 tU, 0–4; Fray Jorge: 0.01 tU, 0–46) and males (Navarino: 0.4 tU, 0–1; Fray Jorge: 0.0 tU, 0–9) from both populations tended to move little between consecutive breeding seasons, usually not crossing an entire territory but breeding in the same or in the nearest vacant nest box. According to the best supported GLMM, there was no effect of sex or population on breeding dispersal distances, and nearly 50% of the total random variance was attributed to intrinsic differences between individuals (Table 1).

Only 35% and 32% of all recaptures represented cases of breeding dispersal in Navarino and Fray Jorge, respectively. In general, nest‐site fidelity (i.e., philopatry or non‐dispersal) was observed in 61% of all female recaptures and in 72% of male recaptures. The best supported model from the hierarchical log‐linear analysis (log‐likelihood ratio test: $\chi_{2}^{2}$ = 2.01, *p *=* *.57) only retained the dispersal status × sex interaction (Wald test *z*_283 _= −1.99, *p *=* *.04), suggesting that the frequency of breeding dispersal differed between the sexes but not between the two populations (see Table 2). Recaptured adults had a minimum age of 2–8 years, but there was no apparent association between breeding dispersal and age class (Figure 4b).

**Table 2:** Frequency of breeding dispersal in female and male thorn‐tailed rayaditos in two peripheral populations

Locality	Dispersal status	Females	Males
Navarino	Nondispersed	32	50
	Dispersed	25	20
Fray Jorge	Nondispersed	53	57
	Dispersed	30	22

### 3.3 Apparent survival

For adult birds, the best model included only six parameters and considered between‐year differences in apparent survival (four temporally varying values; mean ± *SE*: φ_1_: 0.69 ± 0.04, φ_2_: 0.43 ± 0.09, φ_3_: 0.47 ± 0.06, φ_4_: 0.61 ± 0.08) and recapture rate (two values; p_1_: 0.59 ± 0.04, p_2_: 0.77 ± 0.07). According to this model, there were no sex differences in survival probabilities or recapture rates in each population (see Table S2). However, mean apparent between‐year survival was higher in Fray Jorge (0.68 ± 0.0002 *SE*,* n *=* *197) than in Navarino (0.55 ± 0.0008, *n *=* *210). Recapture rates of fledglings were almost equal between the sexes (see *Natal dispersal patterns*), and also suggested higher local survival in Fray Jorge. Vital rates calculated with static life tables indicated that fledgling survival should approximately be 17% in Navarino and 23% in Fray Jorge to maintain both populations constant (see Table S3). Assuming that the contribution of long‐distance immigrants and emigrants to population dynamics is relatively low in comparison with mortality and fecundity (i.e., closed populations), the recovery rate of all marked fledglings would approximately be 18% in Navarino and 26% in Fray Jorge.

### 3.4 Fine‐scale genetic structure

**Figure 5:**
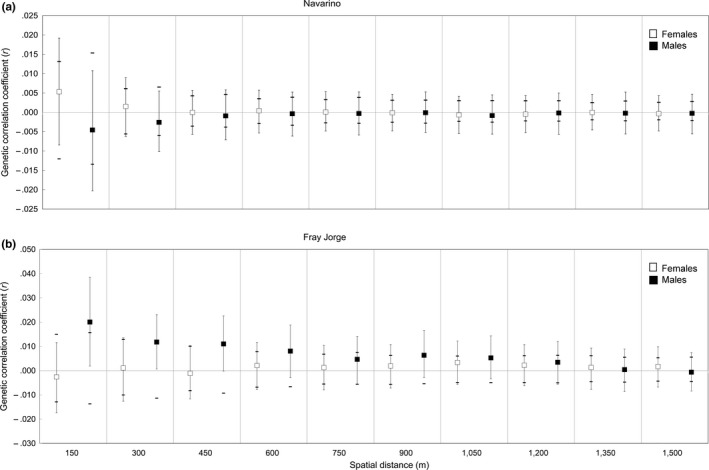
Spatial genetic autocorrelation coefficients (*r*) for increasing distance classes for female and male thorn‐tailed rayaditos in (a) Navarino Island and (b) Fray Jorge National Park. Squares represent correlation coefficients (*r*) with 95% confidence error bars determined by bootstrapping. Solid horizontal dashes represent the 95% CI generated by 1,000 random permutations assuming a random distribution of genotypes (*r* not different from *rp*)

Genetic autocorrelation for increasing distance class sizes for the 2010–2015 dataset (MultiDclass analyzes) showed that neither of the sexes in Navarino departed from a random distribution of genotypes at any of the assessed distance intervals (Figure 5a). Although females in Fray Jorge showed a similar pattern, males exhibited a clear genetic structure at short distances up to 450 m (Figure 5b), with closer individuals being more related than expected by chance (significantly positive *r*‐values). Despite *r‐*values above the 95% CI around *r*_*p*_ up to 600 m, the 95% CI around *r* was significantly different from 0 only up to 450 m (Figure 5b). Analyzes for “snapshot” years showed similar results: No detectable genetic structure in Navarino (females, *n *=* *32; males, *n *=* *31), while in Fray Jorge males showed a significant structure (positive *r*‐values) up to 150 m (females, *n *=* *25; males, *n *=* *25; details not shown).

**Figure 6:**
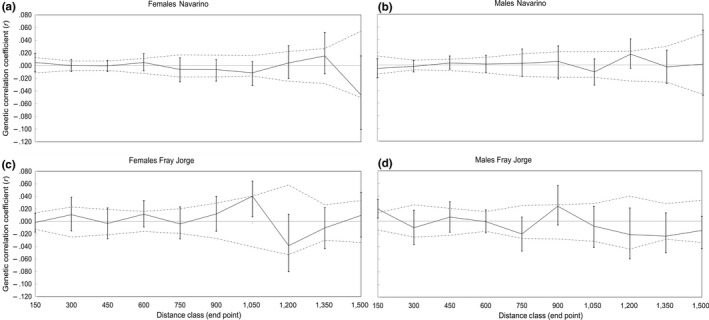
Correlograms showing the genetic correlation (*r*) as a function of spatial distance for thorn‐tailed rayadito. Autocorrelation for distance class sizes of 150 m for (a) females and (b) males in Navarino Island, and (c) females and (d) males in Fray Jorge National Park. Dotted lines represent the 95% CI assuming a random distribution of genotypes (H_0_: *r* not different from *rp*). The 95% confidence error bars about *r* were determined by bootstrapping

Traditional correlograms indicated, again, no detectable genetic structure in Navarino, with correlations for both sexes oscillating between positive and negative values (Figure 6a,b). The nonparametric heterogeneity test revealed no significant differences between the *r*‐values for both sexes at any particular interval (Single‐class *t* ^2^, all *p *>* *.05), and also no sex differences when comparing the whole correlogram (ω = 5.39, *p *=* *.86). Similarly to Multi Dclass analyzes, correlograms for Fray Jorge suggested no genetic structure for females (Figure 6c), but positive and significant *r‐*values for males (Figure 6d). The correlogram for females oscillated between high and low autocorrelation, whereas for males they showed a positive structure at 150 m. The heterogeneity test showed that *r‐*values differed only significantly at the 150 m interval (*t* ^2 ^= 3.03, *p *=* *.04), but the autocorrelation patterns did not reveal significant sex differences (ω = 12.31, *p *=* *.17). Analyzes for “snapshot” years confirmed the same patterns as obtained with the whole 2010–2015 dataset (details not shown).

**Figure 7:**
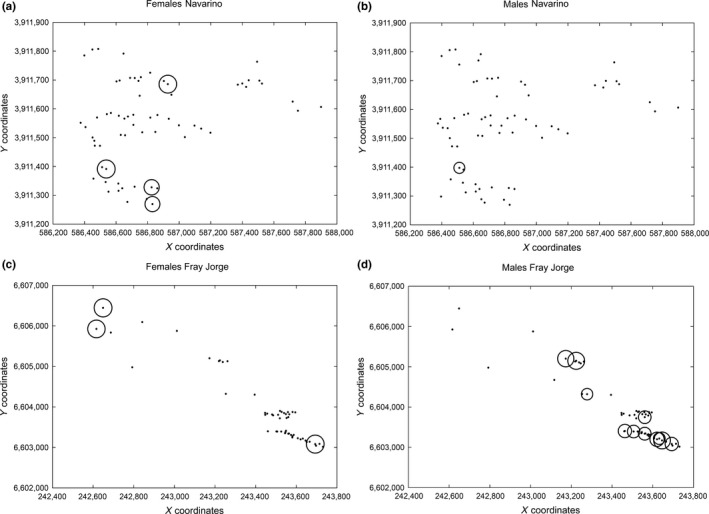
Two‐dimensional autocorrelation analyzes for thorn‐tailed rayadito. Local autocorrelation (*lr*) values were estimated for (a) females and (b) males in Navarino Island, and (c) females and (d) males in Fray Jorge National Park. Only positive and significant *lr*‐values are surrounded by bubbles. Relative bubble size is proportional to the magnitude of *lr*, which ranged from 0.04 to 0.13. All negative *lr‐*values were nonsignificant

For Navarino, the two‐dimensional autocorrelation analyzes revealed significantly positive *lr‐*values for only 5% of the 89 females (*p‐*values for one‐tailed tests: .006–.029) and for only 1% of the 72 males (*p *=* *.047) sampled. The subsets of positively correlated individuals were not geographically clustered, and there were no significant negative *lr‐*values (Figure 7a,b). Although analyzes for Fray Jorge suggested a similar pattern for the 69 sampled females (Figure 7c), with only 4% of significantly positive *lr‐*values (*p = *.012–.021), 17% of all *lr‐*values were significant and positive for the 66 males (*p = *.002–.048), and three small clusters of correlated subsets of males were distinguishable (Figure 7d). Again, there were no significant negative *lr‐*values.

Although Mantel tests are less powerful than autocorrelation analyzes, the observed relationships between pairwise geographic and pairwise genetic distance matrices were consistent with the autocorrelation results. Random permutation tests indicated that correlation coefficients were not different from 0 for females (*r*_*xy *_= .033, *p *=* *.28) and males (*r*_*xy *_= .009, *p *=* *.41) in Navarino, and for females in Fray Jorge (*r*_*xy *_= .035, *p *=* *.26); however, there was a weak, but significantly positive correlation for males in Fray Jorge (*r*_*xy *_= .1, *p *=* *.046).

## 4 DISCUSSION

In this study, we compared local dispersal patterns and microgeographical genetic structure between two peripheral populations of the thorn‐tailed rayadito. These populations differed in terms of habitat fragmentation, breeding density and stress levels of the birds (as measured by baseline plasma corticosterone). The combined results from CMR data and population genetic analyzes showed that local natal dispersal and sex‐specific fine‐scale genetic structure differed markedly between the two populations and exhibited congruent patterns. Local breeding dispersal was much less frequent and involved more restricted movements than natal dispersal and did not vary between the two localities.

The observed patterns of dispersal corresponded with the analyzes of the genetic structure, although the patterns of natal dispersal should be interpreted cautiously given that recaptures of fledglings were in general low. The limited sample size of recruits, particularly in Navarino, might suggest that the spatial scale of the study and the reported dispersal distances are not entirely representative of the actual natal dispersal distances in this population. This is plausible, as the extensive and continuous forest habitat in Navarino could promote higher rates of long‐distance movements compared to Fray Jorge (González & Wink, 2010; Yáñez, 2013). Yet, for several reasons we expect any potential bias in our estimates to be rather low. First, low recovery rates of postfledging individuals are common in dispersal studies regardless of the spatial scale. For instance, fledgling recapture rates in studies encompassing smaller (e.g., Payne, 1991) or larger areas (e.g., Matthysen, 1987; Winkler et al., 2005) than ours, ranged between 3% and 5% (but see Potti & Montalvo, 1991). Second, mortality rates for fledglings in most altricial birds are typically much higher than for adults (Naef‐Daenzer & Grüebler, 2016), implying that many fledglings could have died instead of having dispersed long distances (see e.g., Birkhead, Eden, Clarkson, Goodburn, & Pellat, 1986; Eden, 1987). Our data support this, because estimated fledgling survival rates (assuming no population growth) were three to four times lower than adult apparent survival rates. Third, although we cannot exclude that recruits have dispersed outside the study area, we consider it unlikely that the majority of surviving juveniles moved longer distances than those described here. Instead, missed dispersers in Navarino could have settled near their natal sites using natural cavities for breeding in the mature forest surrounding the study plot. Although fledglings in Fray Jorge could also have moved to other patches around the study area, none of these were farther apart than the longest distances between nest boxes.

### 4.1 Differences in natal dispersal between populations

It has been proposed that the costs and benefits of natal dispersal will be relatively similar for females and males when both sexes select a nest site or establish and defend a territory (Clarke et al., 1997; Greenwood, 1980), potentially leading to no sex‐biased dispersal (Arcese, 1989; Eden, 1987; Enoksson, 1987; Matthysen, 1987). The absence of sex‐biases can also arise when there are no differences in sex‐specific survival rates (Murray, 1967; Waser, 1985; Waser & Jones, 1983), and when movement is not limited by nest site availability or habitat structure and/or saturation (Arlt & Pärt, 2008; Weatherhead & Boak, 1986; Winkler et al., 2005). Conversely, a saturated and fragmented habitat could increase social competition and dispersal frequency/extent (Matthysen, Adriaensen, & Dhondt, 1995; Payne, 1991), generating potentially different responses in each sex depending on the costs and benefits related to dispersal under such conditions (Matthysen, 2012; Starrfelt & Kokko, 2012). Our results support these ideas. The observed distribution of natal dispersal distances did not differ between the sexes in the more continuous environment (Navarino), while females moved longer distances than males in the fragmented and densely populated environment (Fray Jorge). The latter is the most common pattern observed among passerine birds from temperate latitudes (Clarke et al., 1997; Greenwood & Harvey, 1982).

The mechanisms underlying female‐biased natal dispersal in a more heterogeneous and saturated habitat could be manifold, but it has been suggested that dispersing males run higher risks of having to forego breeding due to difficulties finding and defending a suitable breeding site (Payne & Payne, 1993), and due to higher uncertainty about the availability and quality of those sites (Bensch & Hasselquist, 1991; Greenwood, 1980; Winkler et al., 2005). On the other hand, the costs of dispersal for females are expected to be lower, as they typically select among males already settled in a territory (Arlt & Pärt, 2008). Once a subtle sex bias in dispersal behavior has arisen, the social environment could reinforce the asymmetries in dispersal‐related costs experienced by each sex (Payne & Payne, 1993), particularly if adult survival is high, as it is the case in Fray Jorge. Because levels of aggression may increase in saturated and stressful environments, dispersal costs for males will also increase due to the difficulty of establishing a territory in an area with unfamiliar neighbors of the same sex (Payne & Payne, 1993). On the contrary, the social incentive for females returning to the natal area will be low, as their already higher dispersal tendency will decrease the probability of encountering a socially familiar place (Payne & Payne, 1993).

We did not directly assess whether breeding opportunities were more restricted in Fray Jorge compared to Navarino, but CMR data and analyzes of movement patterns support this idea. While most recaptured fledglings from Navarino were yearlings (70%), nearly all recaptures in Fray Jorge were 2‐ to 3‐year‐old birds (93%), suggesting that both females and males delayed their first breeding attempt in the fragmented and densely populated environment (see e.g., Matthysen & Currie, 1996). Although these birds could have remained undetected while breeding in natural cavities inside the study area during their first year, this is unlikely, because bird counts and searches for marked individuals around the nest boxes were carried out on a weekly basis during all years. We cannot rule out the possibility that these birds bred in nearby forest patches outside the study area, but limited breeding dispersal suggests that this is also unlikely.

Monte Carlo simulations also indicated that males in Fray Jorge have more restricted options for finding a breeding territory and/or available mates. Indeed, male movements in Fray Jorge were shorter than expected under a random model, but did not differ from a random‐walk model, which assumes that birds search for any available breeding site starting at their natal nest box and move until an unoccupied area is found (Waser, 1985; Winkler et al., 2005). In contrast, females in Fray Jorge did not occupy the first available vacant site, but traveled longer distances following a more random pattern, possibly resulting from trying to find an established and unpaired territorial male (see Arlt & Pärt, 2008; Wolff & Plissner, 1998). Natal dispersal in Navarino did not differ significantly from either a random model or a random‐walk model, but sample sizes were small, and thus, this is difficult to interpret.

### 4.2 Restricted breeding dispersal in both populations

Theoretical and empirical studies suggest that adult birds, once they established a territory, may pay higher costs of dispersal than younger birds, who have not yet invested in finding and defending a suitable breeding site (Greenwood & Harvey, 1982; Wheelwright & Mauck, 1998) and may thus have less to lose (“asset‐protection” principle; Clark, 1994). As has been found in many passerine species (Greenwood & Harvey, 1982; Paradis, Baillie, Sutherland, & Gregory, 1998), breeding dispersal in rayaditos was indeed less frequent, and dispersal distances much shorter compared to natal dispersal. Nevertheless, breeding dispersal did not significantly differ between males and females or between the two populations.

Breeding dispersal in thorn‐tailed rayaditos was often restricted to movements within the range of a territory (<50 m). The median breeding dispersal distances of females and males were shorter than expected by chance (random model), but not significantly different from a random‐walk process, suggesting that adult birds tended to move to the nearest vacant nest box within their territory, or in some instances, to a contiguous territory. Interestingly, individual identity explained almost half of the observed variation in the number of territories traversed by adult rayaditos. This suggests that breeding dispersal is an individually determined trait, which might partly be genetically determined or may be related to phenotypic quality (Matthysen, 2012). Breeding dispersal may also depend on breeding experience and interactions between the pair members (Harvey et al., 1979, 1984; Payne & Payne, 1993; Valcu & Kempenaers, 2008; Ward & Weatherhead, 2005). These factors should be considered in future studies.

Although the frequency of breeding dispersal was low in both populations, philopatric behavior was more common among males than females, as in many other bird species (Clarke et al., 1997; Greenwood, 1980; Greenwood & Harvey, 1982). This difference could stem from the higher costs of breeding dispersal incurred by males, potentially as a consequence of their greater involvement in the acquisition and subsequent defense of breeding territories (Greenwood, 1980). This indeed seems to be the case in rayaditos, where males are more aggressive than females during territorial intrusions by conspecifics, even though both members of the breeding pair engage in shared territorial defense (Ippi, van Dongen, Lazzoni, & Vásquez, 2017).

The absence of between‐population differences in breeding dispersal patterns in rayaditos could be a by‐product of their dependence on available cavities for reproduction. Similar to other secondary cavity nesters, the availability of nesting cavities appears to be a limiting factor for this species (Botero‐Delgadillo, Poblete, & Vásquez, 2015; Cornelius, 2008; Tomasevic & Estades, 2006), and this would have selected for strong philopatry in adult birds. Because limited opportunities for reproduction reduce the variability in breeding dispersal (Harts, Jaatinen, & Kokko, 2016), present‐day patterns of restricted local movements of breeding adults in both populations might be by‐product of limited access to nesting cavities.

### 4.3 Fine‐scale genetic structure and natal dispersal

Analyzes using the complete 2010–2015 dataset (as well as those based on “snapshot” years) showed that the population genetic structure corresponded to patterns of natal dispersal in both localities. In contrast, breeding dispersal had no influence on microgeographic genetic patterns, as expected, given the limited and less frequent movements of adults compared to fledglings. In Navarino, the absence of a small‐scale population structure is consistent with our observations suggesting nonrestricted, nearly random natal dispersal in both sexes. Similarly, females in Fray Jorge, who dispersed over larger distances that did not differ from a random pattern, also showed no fine‐scale genetic structure. In contrast, males in Fray Jorge, who showed restricted dispersal within forest patches, indeed showed a clear pattern of isolation by distance (Mantel tests), and a positive genetic structure extending to distances up to 450 m (Figure 5b; see Banks, Lindenmayer, Ward, & Taylor, 2005; Double et al., 2005; Peakall et al., 2003; Stow et al., 2001). This distance coincides with the diameter of the largest forest patches in the study area (range: 150–500 m; Figure 2).

The 2D LSA confirmed that the positive genetic structure for males in Fray Jorge was the result of a patchy signal (see Double et al., 2005; Peakall et al., 2003), showing that males were grouped in clusters of local genetic autocorrelation. A closer look at the pairwise genetic distances of males in such clusters revealed that in several cases they consisted of half‐sibs or even full‐sibs or fathers with their sons that ended up breeding within 100–200 m away from each other (see Table S4). The risk of potentially deleterious inbreeding might be reduced because females moved larger distances (e.g., van Dijk et al., 2015). Determining whether inbreeding avoidance is a major cause of female natal dispersal in Fray Jorge is beyond the scope of this study but would be interesting given the apparent isolation of this population of rayaditos and its relatively low genetic diversity (Yáñez, 2013).

In general, the spatial genetic structure of a population reflects a complex interaction between population demography, the social and mating system, and dispersal behavior (Double et al., 2005). In contrast to other studies focused on polygynous or cooperatively breeding birds (e.g., Beck et al., 2008; Double et al., 2005; Temple et al., 2006; Van Dijk et al., 2015), social interactions are not expected to influence the genetic structure in socially monogamous species (Banks & Peakall, 2012; Peakall et al., 2003; but see Foerster et al., 2006). We found no differences in apparent survival between males and females in either of the two populations, so potential effects of sex‐biased mortality can be discarded. Therefore, it seems likely that the observed differences in small‐scale genetic structure between the two populations can be attributed to the different natal dispersal patterns (Banks & Peakall, 2012).

### 4.4 Concluding remarks

Although being capable of colonizing distant oceanic islands (Remsen, 2006), rayaditos show philopatric behavior at the local scale, as described for populations inhabiting patchy environments in central (Vergara et al., 2010) and southern Chile (Cornelius, 2008). Our results provide further evidence of restricted local movements in this forest passerine bird, yet they indicate that natal dispersal patterns vary between populations and might be context‐dependent. Our study also confirms that fine‐scale genetic structure may only be detectable under strong philopatry (see Goudet, Perrin, & Waser, 2002). To determine whether variation in natal dispersal depends on habitat fragmentation, population density, or social interactions either an experimental approach is needed or at least additional populations that differ in these factors should be studied (i.e., “core” populations; see Merrick & Koprowski, 2016). The influence of other environmental stressors such as predator pressure or food availability could also be investigated.

Studies looking at context‐dependent patterns of dispersal and genetic structure can help understand population dynamics within a species' distributional range (Foster, 1999). Peripheral populations with contrasting historical and ecological contexts can be considered natural laboratories for such explorations (Travis & Dytham, 2012). In fact, the observed differences in microgeographic genetic structure in rayaditos might reflect the genetic consequences of population‐specific responses to contrasting environmental pressures near the range limits of their distribution. Because of the unique Chilean geography, many species show similar distributions across a large latitudinal range, and we suggest that a unique opportunity exists here to compare patterns of variation across taxa with varying life history traits and social systems.

## CONFLICT OF INTEREST

None declared.

## AUTHOR CONTRIBUTIONS

EB‐D conceived the idea, conducted the research, analyzed data, and wrote the manuscript. VQ and YP collected part of the data, carried laboratory work, and participated in the design of field and laboratory protocols. EC, SK, AG, and KT carried laboratory work and contributed with ideas for genetic analyzes. EP, BK, and RAV supervised the research and edited the manuscript. All authors gave final approval for publication.

## DATA ACCESSIBILITY

The datasets analyzed during the current study are archived in the Dryad Digital Repository (http://datadryad.org/), doi:10.5061/dryad.7tp60.
