# Supplementary material for: Variation in fine‐scale genetic structure and local dispersal patterns between peripheral populations of a South American passerine bird
Source: Ecol Evol. 2017 Sep 8;7(20):8363–78. doi: 10.1002/ece3.3342 (PMC5648682; doi:10.1002/ece3.3342)
Supplement: Supplementary file 1 [file ECE3-7-8363-s001.docx]

**TABLE S1** Details of 13 polymorphic microsatellite loci and one chromosome-linked marker (Ps/P) for thorn-tailed rayadito. The first eight loci are species-specific markers. M: primer mixes containing four to five primer pairs. T_a_: annealing temperature. C: primer concentration in mix. Size range is in base pairs (bp).

| Locus | Fluorescent dye | M | T_a_ (˚C) | C (μM) | Size range (bp) |
| --- | --- | --- | --- | --- | --- |
| As1 | NED-TTTCCAGTTGTATCTCTCAGCA | 1 | 54 | 0.4 | 217-245 |
|  | GAAGAATGGGATCTAAGAAGTC |  |  |  |  |
| As7 | 6FAM-GCTGGGCTTGCATATTCTTC | 2 | 55 | 0.36 | 213-253 |
|  | TCTTGTTTTGAAGGGAAGTGGA |  |  |  |  |
| As18 | VIC-GGAAGCCATCTTAGGCTGTG | 2 | 55 | 0.6 | 212-220 |
|  | GGGCATAGATGGTTGCTGAT |  |  |  |  |
| As25-1 | PET-GGAGGGTATTTGGCAAGGTT | 2 | 55 | 0.3 | 176-208 |
|  | AGGATGGCTTGCTAGCTGTG |  |  |  |  |
| As25-5 | NED-TGGGTTCAGTATCCTGGAAGA | 2 | 55 | 0.4 | 172-210 |
|  | GAGTTGCTCTTCTCTCCCTCA |  |  |  |  |
| As25-8 | PET-AAGAAGCTCACCCGCTACCT | 1 | 54 | 0.5 | 224-230 |
|  | TGTTGTCGTGCCTGAAGAAG |  |  |  |  |
| As25-10 | VIC-GGAGTTATACCAGTTATAAAGG | 1 | 54 | 0.4 | 142-202 |
|  | TGCTGTTGTCTGGCTAGCA |  |  |  |  |
| As25-14 | 6FAM-TTTCTGCTGCTGGAAAGGTT | 1 | 54 | 0.3 | 177-239 |
|  | GTTCATCCAGGGAGAGTCCA |  |  |  |  |
| Asµ15 ZEST | 6FAM-AATAGATTCAGGTGCTTTTTCC | 3 | 55 | 0.4 | 101-133 |
|  | GGTTTTTGAGAAAATTATACTTTCAG |  |  |  |  |
| CcaTgu23 | PET-CAAGGMYCATGCCAAAATAA | 3 | 51 | 0.8 | 120-161 |
|  | CCCTYCCTCCCTTCAGTTTT |  |  |  |  |
| Tgu05 | NED-CACAGAAAAGTGAGTGCATTCC | 3 | 51 | 0.42 | 252-260 |
|  | TGGGAAAACATCTTTACCATCA |  |  |  |  |
| Tgu06 | VIC-CGAGTAGCGTATTTGTAGCGA | 3 | 51 | 0.34 | 189-219 |
|  | AGGAGCGGTGATTGTTCAGT |  |  |  |  |
| ZF_AC138573 | NED-ATGYCAACTGAAATGTCAAGGT | 3 | 51 | 0.5 | 134-192 |
|  | ATGAGGTCACTGAAAGKTWTAATA |  |  |  |  |
| P2/P8 | 6FAM-CTCCCAAGGA TGAGRAAYTG | 2 | 55 | 0.6 | 356-382 |
|  | TCTGCATCGC TAAATCCTTT |  |  |  |  |

**TABLE S2** Model selection for capture-mark-recapture (CMR) data from two populations (Navarino Island, Nav; Fray Jorge National Park, FJ) of thorn-tailed rayadito. Models’ goodness of fit was assessed using values of Akaike´s Information Criterion corrected for small samples (AICc) where lower values indicated better fit. Model notation: φ = apparent survival; p = recapture rate; FJF = female birds from FJ; FJM = male birds from FJ; NavF = female birds from Nav; NavM = male birds from Nav. Time-dependent variables were denoted with (t), whereas variables that were constant through time were denoted with (.). Equality between populations and/or sexes were denoted with ‘=’, while differences appeared as ‘≠’.

| Model | AICc | Delta AICc | AICc Weights | Model Likelihood | No. Par^*^ | Deviance |
| --- | --- | --- | --- | --- | --- | --- |
| φFJF(t)=φFJM(t)≠φNavF(t)=φNavM(t) pF(t)=pM(t) | 791.91 | 0.00 | 0.52 | 1.00 | 6 | 139.19 |
| φFJF(t)=φFJM(t)≠φNavF(.)=φNavM(.) pF(t)=pM(.) | 795.06 | 3.14 | 0.11 | 0.21 | 5 | 144.39 |
| φFJF(t)≠φFJM(t)≠φNavF(t)≠φNavM(t) pF(t)=pM(t) | 800.36 | 8.45 | 0.01 | 0.01 | 6 | 153.79 |
| φFJF(t)=φFJM(t)≠φNavF(t)=φNavM(t) pF(.)=pM(.) | 805.28 | 13.37 | 0.00 | 0.00 | 6 | 142.12 |
| φFJF(t)=φFJM(t)=φNavF(t)=φNavM(t) pF(t)=pM(t) | 806.58 | 14.67 | 0.00 | 0.00 | 14 | 137.04 |

^*^No. Par: number of parameters in the model.

**TABLE S3** Static life tables for thorn-tailed rayadito assuming no population increase/decrease (*r* = 0) in two populations during 2008-2015 (Navarino Island, Nav; Fray Jorge National Park, FJ). Approximated per capita rates of population growth (*r*) were calculated as ln(R_o_)/G (Stearns, 1992). All survival and fecundity rates for ages 2-8 were estimated from capture-mark-recapture (CMR) data. Numbers in bold represent adjusted vital rates calculated by trial and error until obtaining *r* = 0. *a_x_* = surviving individuals; *l_x_ =* age-specific survival rate; *m_x_ =* age-specific fecundity (mean number of fledglings produced by a female); R_o_ = number of fledglings produced by a female during its entire lifetime (Ʃ *l_x_m_x_*); G = generation time (Ʃ *x* *l_x_m_x_*/R_o_).

| Population | Age | *a_x_* | *l_x_* | *m_x_* | *l_x_m_x_* | *x (l_x_m_x_)* |
| --- | --- | --- | --- | --- | --- | --- |
| Navarino |  |  |  |  |  |  |
|  | 0 | **8076** | **1.000** | 0.0 | 0.000 | 0.000 |
|  | 1 | **1400** | **0.173** | 4.4 | 0.763 | 0.763 |
|  | 2 | 110 | 0.014 | 5.1 | 0.071 | 0.142 |
|  | 3 | 71 | 0.009 | 5.4 | 0.047 | 0.142 |
|  | 4 | 50 | 0.006 | 5.4 | 0.033 | 0.134 |
|  | 5 | 34 | 0.004 | 5.3 | 0.022 | 0.112 |
|  | 6 | 9 | 0.001 | 5.2 | 0.006 | 0.035 |
|  | 7 | 6 | 0.001 | 5.1 | 0.004 | 0.027 |
|  | 8 | 2 | 0.000 | 5.0 | 0.001 | 0.010 |
|  |  |  |  |  | R_o_ = 0.95 | G = 1.40 |
| Fray Jorge |  |  |  |  |  |  |
|  | 0 | **3788** | **1.000** | 0.0 | 0.000 | 0.000 |
|  | 1 | **880** | **0.232** | 3.0 | 0.697 | 0.697 |
|  | 2 | 124 | 0.033 | 3.1 | 0.101 | 0.203 |
|  | 3 | 83 | 0.022 | 3.2 | 0.070 | 0.210 |
|  | 4 | 51 | 0.013 | 3.1 | 0.042 | 0.167 |
|  | 5 | 24 | 0.006 | 3.1 | 0.020 | 0.098 |
|  | 6 | 11 | 0.003 | 3.0 | 0.009 | 0.052 |
|  | 7 | 8 | 0.002 | 3.0 | 0.006 | 0.038 |
|  | 8 | 3 | 0.001 | 2.9 | 0.002 | 0.014 |
|  |  |  |  |  | R_o_ = 0.95 | G = 1.51 |

**TABLE S4** Pairwise relatedness among breeding male neighbours of thorn-tailed rayadito at Fray Jorge National Park (30°38’ S, 71°40’ W), Chile. Relatedness coefficients were estimated for eleven male clusters of 15 individuals that previously showed local positive genetic autocorrelation (*lr*) in a two-dimensional autocorrelation analysis (2D LSA; Double et al., 2005). For every subset showing significant (*p* ≤ 0.05) and positive *lr-*values, we estimated maximum estimates of relatedness (*r*) between a reference male and his 14 nearest neighbours. The *r-*values for each pair of males were transformed into four relationships (U: unrelated; HS: half-siblings; FS: full-siblings; PO: parent-offspring) using the ML-Relate software (Kalinowski, Wagner, & Taper, 2006) to calculate the log-likelihood of these relationships.

| Sample | *lr* | *P* | NN1 | NN2 | NN3 | NN4 | NN5 | NN6 | NN7 | NN8 | NN9 | NN10 | NN11 | NN12 | NN13 | NN14 |
| --- | --- | --- | --- | --- | --- | --- | --- | --- | --- | --- | --- | --- | --- | --- | --- | --- |
| 32 | 0.111 | 0.002 | U | U | U | U | HS | U | U | HS | U | U | U | U | HS | U |
| 20 | 0.109 | 0.003 | PO | HS | U | HS | HS | U | U | HS | HS | U | U | U | U | U |
| 14 | 0.102 | 0.007 | U | U | U | HS | U | U | U | U | HS | PO | PO | U | U | U |
| 49 | 0.068 | 0.02 | U | U | U | U | U | U | U | U | FS | U | U | U | U | U |
| 44 | 0.079 | 0.021 | U | U | PO | HS | U | U | U | HS | U | U | U | U | U | U |
| 63 | 0.067 | 0.026 | U | U | U | U | U | U | HS | U | U | U | U | U | U | U |
| 53 | 0.062 | 0.035 | U | U | U | U | HS | U | U | U | HS | HS | U | U | U | U |
| 26 | 0.063 | 0.037 | U | U | U | U | PO | U | HS | U | HS | U | U | U | U | U |
| 8 | 0.061 | 0.047 | U | HS | U | HS | HS | U | U | U | U | FS | U | U | U | U |
| 43 | 0.054 | 0.048 | U | U | U | U | U | U | U | U | U | U | U | U | HS | U |
| 40 | 0.051 | 0.049 | PO | U | U | U | U | HS | U | U | U | U | U | HS | U | U |
